# Predictors and occurrence of antenatal depressive symptoms in Galle, Sri Lanka: a mixed-methods cross-sectional study

**DOI:** 10.1186/s12884-021-04239-w

**Published:** 2021-11-10

**Authors:** Sage Wyatt, Truls Ostbye, Vijitha De Silva, Prabodha Lakmali, Qian Long

**Affiliations:** 1 Global Health Research Center, Duke Kunshan University, Kunshan, Jiangsu Province China; 2 Duke Global Health Institute, Duke University, Durham, NC USA; 3 Faculty of Medicine, University of Ruhuna, Matara, Southern Province Sri Lanka

**Keywords:** Depression, Mental health, Antenatal care, Obstetric health, Sri Lanka

## Abstract

**Background:**

There is a high prevalence of antenatal depression in low-or-middle-income countries, but information about risk factors in these settings is still lacking. The purpose of this study is to measure the prevalence of and explore risk factors associated with antenatal depressive symptoms in Galle, Sri Lanka.

**Methods:**

This study used a mixed-method approach. The quantitative portion included 505 pregnant women from Galle, Sri Lanka, with health record data, responses to psychometric questionnaires (MSPSS and PRAQ-R2), and antenatal depression screening (EPDS). The qualitative portion included interviews with public health midwives about their experiences and routine clinical practices with women with antenatal depressive symptoms.

**Results:**

Prevalence of antenatal depressive symptoms was 7.5%, highest in women over the age of 30 (13.0%, OR = 3.88, 95%CI = 1.71 – 9.97), with diabetes (21.9%, OR = 3.99, 95%CI = 1.50 – 9.56), or pre-eclampsia in a previous pregnancy (19.4%, OR = 3.32, 95%CI = 1.17 – 8.21). Lower prevalence was observed in the primiparous (3.3%, OR = 0.29, 95%CI = 0.12 – 0.64) employed outside the home (3.6%, OR = 0.33, 95%CI = 0.13 – 0.72), or upper-middle class (2.3%, OR = 0.17, 95%CI = 0.04 – 0.56). Anxiety levels were elevated in depressed women (OR = 1.13, 95%CI = 1.07 – 1.20), while perceived social support was lower (OR = 0.91, 95%CI = 0.89 – 0.93). After multivariable adjustment, only parity (OR = 0.20, 95%CI 0.05 – 0.74) and social support from a “special person” (OR = 0.94, 95%CI = 0.77 – 0.95) remained significantly associated with depressive symptoms. Qualitative findings also identified antenatal health problems and poor social support as risk factors for depressive symptoms. They also identified different contributing factors to poor mental health based on ethnicity, higher stress levels among women working outside the home, and misinformation about health conditions as a cause of poor mental health.

**Conclusions:**

Prevalence of antenatal depressive symptoms in Galle is lower than the recorded prevalence in other regions of Sri Lanka. Risk factors for antenatal depressive symptoms were identified on biological, psychological, and social axes. These variables should be considered when developing future guidelines for mental health and obstetric treatment in this context.

## Introduction

Depression is a psychological disorder with heterogenous symptomatology and causes still being investigated. Currently, depression is estimated to be the third biggest cause of disability worldwide [1]. The postnatal and antenatal periods are especially high-risk times for onset of mental illness. While postnatal depression has received more public awareness and attention, antenatal depression has been shown to be a very strong risk factor. Globally the prevalence of antenatal mental illness varies between 7 to 20% in high income countries, with a wider range from 5 to 50% in low-or-middle-income countries (LMICs). Overall, LMICs have higher rates of antenatal depression on average, but information about antenatal depression in LMICs remains limited [2].

Screening for and prevention of any antenatal health problem is especially important because of the possible adverse consequences for both the mother and the fetus. Some studies have explored a relationship between antenatal depression and poor fetal outcomes, such as increase in stillbirth, low birth weight, and major congenital anomalies [3]. More frequent obstetric visits and overuse of elective caesarean sections are both common in depressed women [4]. Furthermore, antenatal depression is a very strong predictor of post-partum depression, leading to further maternal morbidity after birth [5].

Evidence on risk factors of antenatal depression is inconsistent. While some studies demonstrate significant associations with social determinants, the results are not necessarily replicable in different contexts. Age, parity, and ethnicity have all demonstrated different associations in different studies. Socioeconomic status and education have not been widely shown to be associated with antenatal depression [6]. Support and connection with friends, spouses, and family have been widely characterized as playing a protective role against depression in both general and pregnant populations [7]. Antenatal anxiety is also highly associated with antenatal depression and considered to be a common comorbidity [8], and other chronic health conditions and previous pregnancy complications have also demonstrated a significant association with depression during pregnancy in previous research [9].

Sri Lanka is home to over 20 million people, and currently boasts very strong maternal health indicators. Nearly 100% of all pregnant women nationally receive antenatal care and deliver institutionally, with 95% at public facilities. While private services are available, antenatal healthcare is dominated by public primary care clinics providing care free of charge once every two weeks in addition to outreach services at the patient’s home. Patients needing specialized care are referred to higher level hospitals, staffed by specialist doctors and nurses. Each clinic is supervised by a medical doctor, but most frontline care is conducted by Public Health Midwives (PHMs), from pre-conception through the post-partum period. Health records are organized by the “pregnancy record” system, a physical paper form completed by a midwife on the first antenatal care visit. The patient takes home one copy of the form and is required to bring it to antenatal visits. As of yet, there is no official protocol for screening and treatment for antenatal depression [10].

There has been very little research concerning antenatal depression in Sri Lanka. We therefore carried out a mixed-method study in southern Sri Lanka to investigate prevalence of antenatal depression and examine pregnant women’s demographic, health, and psychometric factors associated with antenatal depression, and to explore the perceptions of antenatal care providers on risk factors pertinent to the communities they serve.

## Methods

### Study context

The Bope Poddala division is a medical administrative area located in Galle district, Southern Province, in the southwest corner of Sri Lanka. The Galle district is relatively strong economically, with only 2.0% of households defined as poor compared to the national average of 3.1% [11]. The most recent census in 2012 indicated that there are 50,331 people living in the Bope Poddala division, with 18,214 females of reproductive age (15 – 39 years old). Buddhist Sinhala people accounted for 96% of the population in the Bope Poddala Division in 2012, though about a quarter of the urban center is Muslim Moor [12].

We used both quantitative and qualitative methods for data collection and analysis. Recruitment of study participants for both the quantitative and qualitative portions of this study took place at all four antenatal clinics within the Bope Poddala Division.

### Quantitative study

#### Data collection

We invited pregnant women who visited any of the four antenatal clinics to join the quantitative study. Participants were recruited over a period of 6 months from March to August 2020. Eligibility criteria included all individuals who were pregnant, visiting an antenatal clinic in the study site, and willing to participate. We used single population proportion formula to calculate sample size, assuming a prevalence of 16.2% based on previous studies, and a confidence interval of 95% [13]. With 209 participants required based on this estimate, our intended enrollment was threefold at 627 individuals overall, allowing distinct analysis of 209 individuals per trimester. However, the study ended due to rising concerns about COVID-19 in the Galle district; this determined the final sample size. Final analysis was only performed on aggregate data, not stratified by trimester, due to less than 209 pregnant women in the first trimester participating.

Quantitative data was collected by research assistants employed by the University of Ruhuna, visiting clinics open once every two weeks and recruiting participants by convenience. The assistants administered two psychometric scales orally to collect data on anxiety (PRAQ-R2) and perceived social support (MSPSS) as well as a screening measure for antenatal depressive symptoms (EPDS). EPDS was originally validated in Sinhala language in 2004 with a cutoff point of >9 and has been used clinically in the postpartum period since 2012 as well as antenatal research [13]. Psychometric characteristics included anxiety and perceived social support. Anxiety was assessed by the Pregnancy-Related Anxiety Questionnaire revised for pregnant women regardless of parity (PRAQ-R2), with subscales for fear of delivery, worry about child’s health, and concern about appearance [14]. Social support was measured by the Multidimensional Scale of Perceived Social Support (MSPSS), with three subscales from family, friends, and a special person [15]. Neither PRAQ-R2 nor MSPSS have been used in Sinhala language or in Sri Lanka previously and were translated only for the purpose of this study.

The assistant also copied information from the patient’s pregnancy record, the portable physical health records carried by the patient, manually onto a paper data collection sheet. Health records provided data on demographic and health characteristics. Because information about ethnicity and religion was not included in the health record, patients were directly asked for this information. The survey with pregnant women and pregnancy record for each individual were matched by a synthetic ID number.

#### Data analysis

The primary outcome of the study was depression as measured by the Edinburgh Postpartum Depression Scale (EPDS). The independent variables used in this study were demographic characteristics, psychometric characteristics, and health characteristics.

For demographic characteristics, education (Primary or Below, Junior Secondary, Senior Secondary, College or Above), employment (Housewife, Outside Home), and social class were determined from self-reported education and occupation in the health records. Social class was extrapolated from this self-reported occupation data base on the Barker and Hall 5-level system, and it was then re-organized for data analysis into four classes (Lower, Lowest, Higher, Highest) by combining the two lowest classes [16]. Continuous age was grouped into categories with approximately equal size (≤25, 26 – 30, ≥31). Religion/ethnicity (Buddhist Sinhala, Other) was collected by orally asking patients. The “Other” category includes minorities who responded Catholic, Muslim, and Hindu as their religion or Tamil, Burgher, and Moor as their ethnicity.

For biological characteristics, information was gathered from the pregnancy record about subfertility (Yes, No), diabetes mellitus (Yes, No), history of low birthweight baby (Yes, No), history of abortion (Yes, No), Body Mass Index (Low, Normal, High), and history of pre-eclampsia (Yes, No). Body Mass Index (BMI) was categorized from a continuous scale into low (≤19), normal (20 – 25), and high (≥26). It was not clear whether diabetes mellitus in the context of the pregnancy record refers to gestational diabetes or chronic diabetes (a local physician explained that this information is extrapolated only from blood sugar levels during antenatal care visits). Trimester was extrapolated from the date of data collection and the reported due date, then categorized into first (0 – 13 weeks), second (14 – 26 weeks), and third (26 – 41 weeks).

Associations between each of the factors and antenatal depressive symptoms was investigated by bivariable and multivariable analysis. A Fischer’s Exact test was used to assess bivariable associations between depressive symptoms (EPDS score >9) and each subject characteristic. A Kruskal Wallis test was performed to assess associations between these two psychometric scales and EPDS-indicated depressive symptoms. Relationships were considered statistically significant if the p-value was less than 0.05. Three models were used in the multivariable analysis, progressively including demographic characteristics, health characteristics, and psychometric characteristics (including data from anxiety and social support surveys). Coefficients and odds ratios for depressive symptoms are presented for each predictor variable. All statistical analysis was performed in Rstudio version 1.3.

### Qualitative study

#### Data collection

This study used an ethnographic approach to elucidate risk factors for antenatal depressive symptoms from the perspective of local frontline health workers. We conducted semi-structured in-depth interviews with public health midwives working in the four clinics in the Bope-Poddala division between June and October 2020. In this jurisdiction, there were a total of 18 PHMs from the four clinics. We invited all PHMs to participate in the interview, but six PHMs showed disinterest in the study or were busy on their routine duties during the data collection period. We interviewed a total 12 PHMs, three from each clinic, to explore the midwives’ perceptions of prevalence and notable risk factors of antenatal depressive symptoms.

Two pre-intern medical doctors (completed coursework but not yet licensed) who were proficient in both Sinhala and English conducted in-depth interviews (IDIs) with participants in Sinhala in a private setting in or around the clinic. They were invited to conduct the interviews on the basis of their language proficiency, experience with IDIs, and personal connection to the research site coordinator in Galle district. There was no prior relationship between interviewers and interviewees, though the pre-intern doctors had more education and carried a higher rank within the medical system than the midwives. The interviews lasted for about 1 hour and were recorded digitally. Initial questions about the prevalence and importance of mental health issues warranted short responses, while all other questions were open-ended. Participants were first asked the general question “what do you think leads to depressive symptoms,” then followed by probes into how demographics, social support, and physical health impact the condition. The research team developed the topic guide for the interviews, and a local collaborator of the project discussed the study objective and questions in the topic guide with both interviewees. The interview guides were not modified across the study period.

#### Data analysis

Recordings were transcribed directly into English while listening to the Sinhala recording by the moderator who performed the interviews. The moderator then wrote a summary of each transcript in addition to the verbatim translation. The second moderator then verified the translation by reading the transcript and listening to the recording simultaneously. English transcripts and summaries were then shared within a secure REDcap network between the authors after the local partners had completed data collection.

Transcripts were read line by line to identify key concepts and develop a coding scheme. Coding was guided by the theories of applied thematic analysis. After familiarization with the transcripts, structural codes were developed based on the moderator’s guide and emergent themes were identified. This coding scheme was further refined after memo writing on uncoded data, preliminarily coded data, and re-coded data. There was no inter-coder verification process performed, but the authors discussed and verified preliminary findings with research collaborators residing and practicing medicine in Sri Lanka. All transcripts were coded in NVivo 12.

## Results

### Quantitative findings

#### Prevalence of depressive symptoms and associated characteristics

A total of 505 participants were recruited for the quantitative portion. Around one fourth of pregnant women were younger than 25 years old, and the those aged 26-30 and those older were similar in proportion, accounting for 37.2 and 36.4%, respectively. Age of participants ranged from 16 to 43 years old. Few women had education at primary level or below, half of women had junior secondary education and others had senior education or above. More than half were full time housewives, and 12.9% were grouped into the lowest social class. Only 10.5% were in the first trimester of pregnancy, and women in the second and third trimester accounted for 45% of total participants, respectively. Less than half were nulliparous, and ethnic and religious minorities accounted for 22.6%. Most were healthy and had no risk conditions. The majority had a normal BMI, with 6.3% reporting diabetes and 6.1% reporting a history of pre-eclampsia.

The scales of PRAQ-R2 and MSPSS were used to measure anxiety of pregnant women and perceived social support. Both PRAQ-R2 (alpha = 0.715) and MSPSS (alpha = 0.861) had strong internal validity. The PRAQ-R2 mean score of 15.5/50 indicated overall low levels of anxiety and the mean MSPSS score of 72.6/84 indicated overall satisfactory social support in this community. The most common cause of the anxiety in the study participants was anxiety about delivery, and the strongest social support came from family (Table 1).

**Table 1:** Participant characteristics extracted from pregnancy records and psychometric questionnaires

	Total n(%)
Demographic and socioeconomic	
**Age**	
≤25	133(26.3)
26 – 30	188(37.2)
≥31	184(36.4)
**Education**	
Primary or Below	23(4.5)
Junior Secondary	252(49.9)
Senior Secondary	171(33.9)
College or Above	59(11.7)
**Employment**	
Outside Home	197(39.0)
Housewife	308(61.0)
**Social class**	
Highest	73(14.3)
Higher	172(34.1)
Lower	195(38.6)
Lowest	65(12.9)
**Trimester**	
1	53(10.5)
2	229(45.3)
3	223(45.2)
**Parity**	
Primiparous	211(41.8)
Multiparous	294(58.2)
**Religion/Ethnicity**	
Buddhist Sinhala	391(77.4)
Other	114(22.6)
Health	
**Subfertility**	
Yes	38(7.5)
No	467(92.5)
**Diabetes mellitus**	
Yes	32(6.3)
No	473(93.7)
**BMI**	
Low BMI	130(25.8)
Normal BMI	288(57.1)
High BMI	86(17.1)
**History of LBW baby**	
Yes	38(7.5)
No	467(92.5)
**History of abortion**	
Yes	61(12.1)
No	444(87.9)
**History of pre-eclampsia**	
Yes	31(6.1)
No	474(93.9)
Psychometric	
**Anxiety total**	15.5(5.0)
10 – 50 mean(sd)	
**Anxiety about delivery**	6.5(3.3)
3 – 15 mean(sd)	
**Worry about baby’s health**	5.0(1.7)
4 – 20 mean(sd)	
**Concern about appearance**	4.0(1.9)
3 – 15 mean(sd)	
**Social support total**	72.6(11.8)
12 – 84 mean(sd)	
**Support from family**	25.9(3.6)
4 – 28 mean(sd)	
**Support from friends**	21.5(8.0)
4 – 28 mean(sd)	
**Support from a special person**	25.3(4.5)
4 – 28 mean(sd)	

The overall prevalence of serious depressive symptoms was 7.5%, as 38 out of the 505 women scored above the diagnostic cutoff (>9) (Table 2). Prevalence of depressive symptoms was higher among pregnant women above 30 years old, multiparous women, those who were housewives, or from low social class. There was a higher prevalence in lower educated populations, but this was not found to be statistically significant.

**Table 2:** Prevalence of antenatal depression (as measured by EPDS) across demographic and health characteristics

	Depression prevalence n(%)	*p*-value*
**Total**	38(7.5)	
Demographic and socioeconomic		
**Age**		**0.002**
≤25	7(5.3)	
26 – 30	7(3.7)	
≥31	24(13.0)	
**Trimester**		0.092
1	5(9.4)	
2	11(4.8)	
3	22(9.8)	
**Parity**		**0.002**
Primiparous	7(3.3)	
Multiparous	31(10.5)	
**Employment**		**0.009**
Outside work	7(3.6)	
Housewife	31(10.1)	
**Religion/Ethnicity**		1.000
Buddhist Sinhala	30(7.7)	
Other	8(7.0)	
**Education**		0.076
Primary or Below	3(13.0)	
Junior Secondary	25(9.9)	
Senior Secondary	8(4.7)	
College or Above	2(3.4)	
**Social class**		**0.003**
Highest	5(6.8)	
Higher	4(2.3)	
Lower	21(10.8)	
Lowest	8(12.3)	
Health		
**Subfertility**		0.516
Yes	4(10.1)	
No	34(13.7)	
**Diabetes mellitus**		**0.006**
Yes	7(21.9)	
No	31(6.6)	
**History of LBW baby**		0.055
Yes	6(15.8)	
No	32(6.9)	
**History of abortion**		0.201
Yes	7(11.5)	
No	31(7.0)	
**BMI**		0.234
Low BMI	7(5.4)	
Normal BMI	21(7.3)	
High BMI	10(11.6)	
**History of pre-eclampsia**		**0.022**
Yes	6(19.4)	
No	32(6.8)	

^*^ Bolded values with *p*<0.05

The highest prevalence of depressive symptoms was observed in those with diabetes mellitus, at 21.9% (Table 2). There was also higher prevalence of depressive symptoms reported among those with a history of complication or abortion in a previous pregnancy. Meanwhile, prevalence was lowest in those with low or normal BMI, and those with a history of subfertility.

Anxiety levels self-reported via PRAQ-R2 were higher among women with serious depressive symptoms (EPDS >9) than among those without. Mean PRAQ-R2 subscore for concern about appearance differed by depressive symptom status to a smaller extent than the other subscales. Social support measured by MSPSS was overall lower in depressed women. This association was robust for all subscales (*p* <0.001) (Table 3).

**Table 3:** Associations between psychometric variables and antenatal depression as measured by EPDS

	Depressed mean(SD)	Not Depressed mean(SD)	*p*-value*
Anxiety total	19.3(6.4)	15.2(4.8)	**<0.001**
Anxiety about delivery	7.8(3.6)	6.4(3.3)	**0.013**
Worry about baby’s health	6.7(3.0)	4.9(1.5)	**<0.001**
Concern about appearance	4.4(2.2)	4.0(1.9)	0.189
Social support total	56.9(18.9)	73.9(10.0)	**<0.001**
Support from family	21.4(6.3)	26.3(3.0)	**<0.001**
Support from friends	16.1(8.7)	21.9(7.7)	**<0.001**
Support from a special person	19.5(8.4)	25.8(3.6)	**<0.001**

* Bolded values with *p*<0.05

#### Multivariable adjustment models

Pregnant women over 30 (OR 3.88; 95%CI 1.71, 9.97) were more likely to report antenatal depressive symptoms than women age 26 to 30, even after adjusting for other demographic variables (OR 3.15; 95%CI 1.31,8.53) and health variables (OR 2.65; 95%CI 1.05, 7.35). Primiparous women (OR 0.29; 95% CI 0.12, 0.64) and employed women (OR 0.33; 95%CI 0.13, 0.72) were less likely to experience antenatal depressive symptoms, compared to multiparous women and housewives. After adjusting for other demographic characteristics, the difference between employed women and housewives was statistically significant (OR 0.38; 95%CI 0.13, 0.98), but the difference between primiparous and multiparous women was not statistically significant. After adjusting for demographic and socio-economic characteristics, health status and psychometric variables, multiparity showed statistically significant association with depressive symptoms (OR 0.20; 95%CI 0.05, 0.74). In the univariate analysis, higher social class was the only group found to have significantly lower odds of antenatal depressive symptoms than the lowest social class (OR 0.17; 95%CI 0.04, 0.56), while no statistically significance was found in multivariate analysis. There were no significant associations with depressive symptoms among women by educational groups, religion/ethnicity groups, or by trimester (Table 4).

**Table 4:** Bivariable and multivariable regression analysis of demographic, health, and psychometric variables as predictors of antenatal depression (as measured by EPDS). Bivariable and multivariable logistic and linear regression (Odds Ratio, 95% CI)^a^

	Bivariable	Model 1: Demographic^b^	Model 2: Demographic^b^ + Health	Model 3: Demographic^b^ + Health + Psychometric
Demographic and socio-economic				
**Age**				
≤25	1.43(0.48,4.29)	1.07(0.32,3.54)	1.31(0.38,4.76)	2.02(0.49,8.38)
26 – 30	1	1	1	1
≥31	**3.88(1.71,9.97)**	**3.15(1.31,8.53)**	**2.65(1.05,7.35)**	1.65(0.56,5.32)
**Trimester**				
1	1	1	1	1
2	0.48(0.17,1.60)	0.63(0.20,2.24)	0.58(0.18,2.11)	0.43(0.11,1.95)
3	1.05(0.41,3.26)	0.91(0.46,4.48)	1.24(0.42,4.28)	1.07(0.31,4.44)
**Parity**				
Primiparous	**0.29(0.12,0.64)**	0.43(0.15,1.07)	0.41(0.14,1.14)	**0.20(0.05,0.74)**
Multiparous	1	1	1	1
**Employment**				
Outside work	**0.33(0.13,0.72)**	**0.38(0.13,0.98)**	0.40(0.13,1.06)	0.45(0.13,1.39)
Housewife	1	1	1	1
**Religion/Ethnicity**				
Buddhist Sinhala	1	1	1	1
Other	0.91(0.38,1.95)	0.72(0.26,1.81)	0.70(0.25,1.80)	0.75(0.22,2.29)
**Education**				
Primary or below	4.28(0.66,34.28)	2.11(0.22,23.5)	2.86(0.29,33.69)	0.29(0.02,5.37)
Junior Secondary	3.14(0.90,19.86)	1.12(0.22,8.58)	1.28(0.24,10.11)	0.55(0.09,5.02)
Senior Secondary	1.40(0.34,9.45)	0.53(0.10,4.14)	0.64(0.12,5.19)	0.40(0.06,3.46)
College or Above	1	1	1	1
**Social class**				
Highest	0.52(0.15,1.66)	0.60(0.15,2.32)	0.66(0.15,2.73)	0.57(0.09,3.26)
Higher	**0.17(0.04,0.56)**	0.27(0.06,1.14)	0.33(0.07,1.49)	0.48(0.08,2.94)
Lower	0.86(0.37,2.16)	0.91(0.33,2.82)	1.02(0.35,3.35)	1.09(0.30,4.71)
Lowest	1	1	1	1
Health				
**Subfertility**				
Yes	1.50(0.43,4.04)		1.78(0.43,5.87)	1.70(0.35,6.56)
No	1		1	1
**Diabetes mellitus**				
Yes	**3.99(1.50,9.56)**		1.81(0.58,5.23)	1.72(0.44,6.21)
No	1		1	1
**History of LBW baby**				
Yes	2.55(0.91,6.18)		1.10(0.35,3.06)	1.56(0.42,5.21)
No	1		1	1
**History of abortion**				
Yes	1.73(0.67,3.91)		1.03(0.35,2.70)	0.84(0.24,2.61)
No	1		1	1
**BMI**				
Low BMI	0.72(0.28,1.67)		0.82(0.29,2.14)	0.62(0.17,1.97)
Normal BMI	1		1	1
High BMI	1.67(0.72,3.62)		1.19(0.47,2.83)	1.66(0.56,4.71)
**History of pre-eclampsia**				
Yes	**3.32(1.17,8.21)**		1.96(0.60,5.76)	2.67(0.58,10.54)
No	1		1	1
Psychometric				
**Anxiety total**	**1.13(1.07,1.20)**			
**Delivery**	**1.12(1.02,1.22)**			1.28(0.98,1.30)
**Baby’s health**	**1.48(1.29,1.71)**			1.14(0.92,1.41)
**Appearance**	1.09(0.93,1.24)			0.94(0.72,1.16)
**Social support total**	0.91(0.89,0.93)			
**Family**	**0.79(0.74,0.85)**			0.94(0.92,1.02)
**Friends**	**0.93(0.89,0.96)**			1.13(0.83,1.06)
**Special person**	**0.83(0.79,0.87)**			**0.94(0.77,0.95)**

^a^ Bolded values indicate statistical significance with 95% confidence interval (CI)

^b^ Demographic and socioeconomic factors

Pregnant women with diabetes mellitus (OR 3.99; 95%CI 1.50, 9.56) and a history of pre-eclampsia (OR 3.32; 95%CI 1.17, 8.21) were found to have significantly higher odds of antenatal depressive symptoms, though this association was reduced after adjustment for other variables (Table 4).

After adjustment for all variables, only support from the “special person” remained significantly associated with depressive symptoms (OR 0.94; 95%CI 0.77, 0.95*)*. The relationship between antenatal depressive symptoms and self-reported anxiety was reduced after adjustment (Table 4).

### Qualitative findings

All 12 PHM interviewees were female, and their age varied between 35 years old and 64 years old. Half of them had over ten years work experience in the area of midwifery, ranging from 6 to 30 years total. When asked how often they see patients with antenatal depressive symptoms, some (*n*=5) said that such patients were a common occurrence while others (*n*=7) claimed they were extremely rare, only seeing a few across their whole careers. Among those who said it was a common occurrence, estimates of prevalence ranged from 1 to 5%. Midwives assessed depressive symptoms through subjectively observing and talking with patients, as no screening tool was currently used. They described that depressive symptoms were often identified by changes in mood, talkativeness, or personal hygiene.

#### Topic 1: Demographic, socio-economic and cultural factors

While unusually young or old age was mentioned as a possible risk factor for depressive symptoms, midwives provided no further explanation as to why. Most midwives explained that having more children at home was associated with high workload and stress of taking care of other children, and thus women have less time for taking care themselves. In addition, some midwives commented on the connection between religion/ethnicity and antenatal depressive symptoms. A few midwives thought the Muslim Moor minority community often has many children, which might contribute to poor mental health. On the other hand, a few midwives expressed Muslim Moor pregnant women often have better support from the extended family to cope with challenges during pregnancy and these midwives perceived better mental health status among Muslim Moor pregnant women than among Sinhala pregnant women.

“*Also from the mothers I have met, teenage mothers and elderly mothers have a greater tendency to develop depression/anxiety… My service area is a Muslim community and the major challenge that they face is poor spacing between children and having many kids to deal with.” (IDI, PHM 3)*

*“Those with unexpected pregnancies, poor spacing between children and the ones with many kids are the type of women who typically show these symptoms. The latter type of women find themselves unable to cope up with the workload and develop psychological stress that ultimately develop in to depression.” (IDI, PHM 1)*

*“Specially primi mothers have lot of uncertainty about the future of their pregnancy, as they have not experience the process of delivering a baby before. They face lot of mental distress with the new experiences during the pregnancy” (IDI, PHM 6)*

*“Being a primi also is a risk factor for antenatal mental health problems. Sometimes there are mothers who do not have any additional risk factors but may get antenatal mental health issues.” (IDI, PHM 10)*

*“Mental health problems are common in multiparous mothers than primi mothers sometimes, they often face problems on attending to care of current pregnancy while taking care of other children at home specially if the gap between the pregnancies is very low and the child/children at home are very young. The more the number of children they have at home the more the tendency to have mental health problems... Major challenges are the work overload at home and lack of support from others. This is common in Sinhalese mothers as mothers in Muslim community get a significant support from the extended family. (they live with their mother, sisters aunties therefore they have less responsibilities)” (IDI, PHM 2)*

Midwives also commented on the impact of finances and employment on mental health of pregnant women. Most suggested that financial struggles were one of the greatest challenges faced by their community. Many midwives simply referred to money problems as a stress by itself, while one further explained that poverty is stressful because women are unable to care for their health needs adequately during pregnancy and conflict over finances strains the relationship between husband and wife. Some midwives suggested (in contrast with the quantitative findings) that employed women are more likely to be depressed than housewives. They thought that women who work can struggle with lack of time to fulfill responsibilities and higher stress from their job that can ultimately develop into depression. On the other hand, one midwife commented that housewives may also face issues with stress from their home duties, especially if they must face them alone and feel isolated.

*“Some pregnant mothers face socioeconomic problems such as poverty, domestic violence and other relationship issues. Financial problems play a major role. Some do not have enough money to get a proper nutritious diet. Some face difficulties in buying their needs even the vitamin tablets we prescribe.” (IDI, PHM 6)*

*“Mental stress at the workplace and extramarital affairs of the husband are some common examples that we find as route cause when digging in to the history of pregnant mothers with mental health issues.” (IDI, PHM 11)*

*“Their biggest worry is that they can’t spend lot of time outside the house because of the responsibilities that they have at home. If the woman is employed that is also a challenge to the pregnant woman.” (IDI, PHM 10)*

*“When the pregnant woman has to deal with the workload at home, alone, she feels psychologically stressed*.” *(IDI, PHM 9)*

*“Antenatal mental health problems are common in educated females and those who are going to work.”(IDI, PHM 5)*

#### Topic 2: Health related factors

Most of the midwives commented on health conditions as major contributing factors to antenatal depressive symptoms, specifically those diagnosed with diabetes. They said having diabetes while pregnant could make life difficult because women are expected to undergo more medical procedures, take medicines, and be more mindful of their health. Generally, the midwives said that women are constantly worrying about their own health and the health of their baby and added stress of having a condition or a history of complications could lead to poor mental health. Many midwives further explained that this worry does not necessarily come from a health problem directly, but by misinformation from lay people or the internet which leads pregnant women to believe they have a disease or that the risks of the disease are more serious than in reality.

*“if a pregnant mother has gestational diabetes, that mother have to control the diet, do frequent blood investigations, and they need to admit to the hospital several times for checkups, sometimes they have to take medicines or insulin. All these situations are stresses for them and can lead to mental health issues.” (IDI, PHM 5)*

*“For instance when a mother with gestational diabetes get to know the fact that diabetes mellitus can ultimately lead to heart disease, limb amputations and so on, they start worrying constantly whether a similar thing would happen to them” (IDI, PHM 3)*

*“In gestational diabetes, mothers have to control the diet. Mothers may think that restriction of the diet may affect the nutrition of the baby. She may think that the baby will not gain enough weight.” (IDI, PHM 10)*

*“When a woman with a significant past medical history gets pregnant what first comes to her mind is that her medical condition would have adverse effects on her baby. She keeps on worrying about the issue and numerous opinions from lay people create even a worse picture in her mind making her more anxious and worried. I think that if these pregnant women are properly acknowledged about their medical condition these misconceptions won’t occur and the influence on her mental health would be minimal… As I said they receive various informal/lay inputs from friends,relatives and neighbours and I have seen pregnant mothers suffering severely due to false information.” (IDI, PHM 1)*

*“Nowadays pregnant mothers use internet and find details about their diseases and the incomplete knowledge that they get result in unnecessary worry and that can affect their mental health.” (IDI, PHM 2)*

#### Topic 3: Social support and pregnancy-related anxiety

Most midwives observed the main sources of support coming from the husband and other female family members, with the husband’s role growing in importance in recent years. Many challenges relating to the husband-wife relationship were mentioned as risk factors for depressive symptoms, including extra-marital affairs, substance use disorders, heavy workload, disrespect during decision making, or even domestic violence. All midwives strongly emphasized that poor social support is a key predictor for mental health issues, and strong social support an important protective factor. Anxiety was generally not a familiar concept to the participants, with 6 out of 12 becoming confused or responding they had never heard of anxiety before when asked about the prevalence and typical symptoms. However, many described anxiety as a symptom or precursor of depression.

*“As I haven’t seen a mother with antenatal anxiety, I’m not sure about this. But I think antenatal anxiety lead to depression if not treated properly at initial stages.” (IDI, PHM 12)*

*“I think they are related. Sometimes both conditions share the same features such as excessive worry. According to my level of understanding anxiety comes first and patients come with full blown depressive symptoms if we miss them at the initial stages.” (IDI, PHM 5)*

*“Living at in laws’ house after marriage is the major challenge our mothers face. They feel left out and conflicts with mother-in-law are very common. Along with this pressure, if the husband also shows poor interest in seeking wife’s support in decision making, considering her as a patient, the matter gets worse… Compared to the old days when mother was the main care taker of the pregnant woman, recently husbands have been more caring and more interested in their pregnant wives’ health problems.” (IDI, PHM 1)*

*“I think lack of support from the husband and other family members is a main problem that these mothers face. When she is not receiving enough support, she will feel unsecured and will be feeling helpless. These emotions can lead in mental health problems in antenatal mothers” (IDI, PHM 10)*

*“Husbands are the main care takers, but mother-in-law also takes good care of the pregnant mother in such instances.” (IDI, PHM 11)*

*“Sometimes the problems of the family causes mental health problems in antenatal period. And sometimes lack of support from the family members also lead to mental distress in these mothers… if they have support from their husband, mother, mother in law, sisters its very helpful for them.” (IDI, PHM 2)*

## Discussion

### Interpretation

The prevalence of antenatal depressive symptoms reported here at 7.5% was lower than many previous reports from other countries, ranging from 15 to 65% [17]. The prevalence found in this area of Sri Lanka was also lower than a previous prevalence estimate in Anuradhapura, North Central Province, Sri Lanka, of 16.2% [13]. There may be regional differences in the experience and frequency of mental health issues, as prevalence of postpartum depressive symptoms found in a previous study conducted in the Bope Poddala district was 7.8%, only 0.3% higher than the prevalence in this study [18]. The wide range of estimated prevalence by midwives in this community is probably due to the lack of a standardized screening tool for depressive symptoms during pregnancy. While midwives may have learned to notice common symptoms of depression due to their official training for EPDS screening and treatment of postpartum depression, this has not led to consistent clinical practice antenatally [10].

An association between high parity and depressive symptoms was strongly supported by the quantitative data. Midwives revealed further in IDIs that higher parity may lead to poorer mental health because of a busy schedule taking care of more children and inconvenience for seeking proper healthcare during pregnancy. The midwives’ description of ethnic minorities desiring and having more children is consistent with national level survey data [19]. One previous study performed in Colombo, Sri Lanka also demonstrated a significantly lower prevalence of major depressive symptoms in the ethnic minority population [20], supporting the present midwives’ assertation that ethnic minorities have better mental health despite higher parity due to stronger social support. However, there was no relationship between depressive symptoms and ethnicity/religion observed in this study. It should also be noted that while Tamils and Moors are minority groups in this area of Sri Lanka, their situation may be different in the areas further North where they may be the majority.

Housewife status as a risk factor for depressive symptoms, as indicated by the health records, contrasts with the information from in-depth interviews. A previous study in Sri Lanka measuring major depression demonstrated that employment at a job was a risk factor for depression, and suggested this was due to higher stress, especially for lower-income women, parallel with midwife explanations in this study [20]. IDIs revealed that insufficient money and financial troubles may contribute to poor mental health, and hence more money earned through employment may reduce risk of depressive symptoms, corroborating present quantitative evidence of higher prevalence of depressive symptoms in lower socioeconomic classes. Further, employment remained significantly protective against depressive symptoms even after adjustment for social class and other demographic and socioeconomic variables. One possibility proposed by previous research outside of Sri Lanka is that pregnant women who report employment as “housewife” have a higher prevalence of depressive symptoms because they have stopped working due to the pregnancy. Patients may be experiencing additional psychological problems after termination or pause of their work if they become isolated from their social support system at the job and have lessened economic independence or wish to work but were forced to stop [21, 22]. When considering employment status was not found to be a predictor for postpartum depression in a previous study conducted in the same community [18], the special conditions of stopping work because of pregnancy may be a possible explanation.

While midwives reported in IDIs that overall poorer health contributed to poor mental health, medical record data suggested that only diabetes and a history of pre-eclampsia were significantly associated with depressive symptoms. The highest prevalence of depressive symptoms was also reported in the oldest age group, which could be due to greater risk of pregnancy complications at an older age [23]. While the second multivariable model showed reduced significance of diabetes and pre-eclampsia as risk factors, older age remained robustly associated with depressive symptoms. Midwives suggested that in some cases the association between health problems and mental health is not driven by an underlying biological cause but rather stress over misinformation and sensationalizing the disease. Age in this case may be considered an independent risk factor from associated health problems, contributing to depressive symptoms through higher stress rather than mediated through another specific physical condition.

An association between diabetes and major depression is well established [24]. There is also very strong evidence for a relationship between gestational diabetes and antenatal mental disorders which directly supports the findings in this study [25]. While previous literature has focused on an underlying biological connection between diabetes and depression, the qualitative data in this study suggested that the link between the two disorders may be social. The added stress of additional medical treatments for diabetes plus the worry about the baby’s health was considered a noteworthy cause of antenatal depressive symptoms by the midwives. Likely, this comorbid relationship is very complex and contributing factors have both a biological and social basis.

Both parts of this study suggested strong correlations between social support and antenatal depressive symptoms. This association has been widely reported [26]. Previous studies are consistent with present findings in that MSPSS score in pregnant women was lower for friends than any other source [27]. IDIs clarified that the “special person” as identified on the MSPSS most likely refers to the husband in this context. Quantitative findings that the greatest difference in average social support scores between depressed and non-depressed was from the special person and the significant difference withstanding adjustment from the all-variable model added further evidence to midwives’ assertion that support from the husband is most important.

Previously observed associations between antenatal depression and anxiety were confirmed in this study [8]. While there was a modest positive association between anxiety about body imagine (PRAQ-R2 subscale 3, Concern about Personal Appearance), the relationship in this population was not robust, parallel to previous studies [28]. While the interviewed midwives had very little knowledge of anxiety during pregnancy, they did implicitly suggest that there is an association between antenatal depression and antenatal anxiety by describing anxiety as a symptom of depression. Lack of knowledge is probably due to lack of official guidelines on mental health care during the antenatal period and on anxiety in obstetric practice generally [10].

### Strengths and limitations

This study adds to a growing body of literature on the causes of mental health issues during the maternal care period in Sri Lanka. All data entries for all participants were complete due to the interview-style data collection, and there were no other missing data. The mixed-methods design also allowed for deeper analysis of the results pertaining specifically to this community. However, some of the data, especially relating to physical health, were inconsistently recorded, likely due to problems with the initiation of the pregnancy record. While a condition was considered to be present if it was indicated anywhere on the record, there was potential for overreporting relating to the physical health variables if an indication was a mistake. The lack of information clarity on health records distinguishing mothers with chronic diabetes from those with gestational diabetes poses a further limitation in the analysis for physical health. Also, though the prevalence of COVID-19 was low in Galle during the majority of the study period, social distancing guidelines could have led to some selection bias, as pregnant women were only selected from clinics while an increasing number of patients chose in-home antenatal visits as a safer alternative. Lack of details in qualitative data on how age and finances may affect antenatal mental health should be explored further in future research. Given the cross-sectional nature of this study, there is a possibility for reverse causality between depressive symptoms and the risk factors presented here, so conclusions should be interpreted with caution. Results also may not be generalizable to other regions of Sri Lanka, especially those with different religious and ethnic makeups. The definition of depressive symptoms as used in this study should not be considered a clinical diagnosis, as EPDS is only a screening questionnaire, and not used clinically for antenatal depression in Sri Lanka. PRAQ-R2 and MSPSS are also not previously validated for use in Sinhala language in Sri Lanka, so translation errors and poor reliability are possible. The psychometric characteristics of these survey tools in this population should be explored in future studies to better understand our data.

## Conclusions

While this community has a comparatively low prevalence of antenatal depressive symptoms, the condition remains an underrecognized issue by the health system. The biopsychosocial model of disease is highly applicable to antenatal depressive symptoms in this setting and should be considered when designing interventions. Future research should investigate how these risk factors may act as mediators for other risk factors, and how they change over time.

## Data Availability

The datasets generated and analyzed during the current study are not publicly available due to the risk of compromising personal health information and privacy of the participants but are available from the corresponding author on reasonable request.

## Abbreviations

LMIC: Low-or-Middle-Income Country
PHM: Public Health Midwife
PRAQ-R2: Pregnancy Related Anxiety Questionnaire, Revision 2
MSPSS: Multi-dimensional Scale of Perceived Social Support
EPDS: Edinburgh Postpartum Depression Scale
IDI: In-Depth Interview

## References

[1] Park LT, Zarate CA (2019). Depression in the primary care setting. N Engl J Med.

[2] Biaggi A, Conroy S, Pawlby S, Pariante CM (2016). Identifying the women at risk of antenatal anxiety and depression: a systematic review. J Affect Disord.

[3] Räisänen S, Lehto SM, Nielsen HS, Gissler M, Kramer MR, Heinonen S (2014). Risk factors for and perinatal outcomes of major depression during pregnancy: a population-based analysis during 2002–2010 in Finland. BMJ Open.

[4] Andersson L, Sundström-Poromaa I, Wulff M, Åström M, Bixo M (2004). Implications of antenatal depression and anxiety for obstetric outcome. Obstet Gynecol.

[5] Leigh B, Milgrom J (2008). Risk factors for antenatal depression, postnatal depression and parenting stress. BMC Psychiatry.

[6] Lancaster CA, Gold KJ, Flynn HA, Yoo H, Marcus SM, Davis MM (2010). Risk factors for depressive symptoms during pregnancy: a systematic review. Obstet Gynecol.

[7] Milgrom J, Hirshler Y, Reece J, Holt C, Gemmill AW (2019). Social support-a protective factor for depressed perinatal women?. Int J Environ Res Public Health.

[8] Falah-Hassani K, Shiri R, Dennis C (2017). The prevalence of antenatal and postnatal co-morbid anxiety and depression: a meta-analysis. Psychol Med.

[9] Brown HK, Wilton AS, Ray JG, Dennis C, Guttmann A, Vigod SN (2019). Chronic physical conditions and risk for perinatal mental illness: a population-based retrospective cohort study. PLoS Med.

[10] Hemachandra N. Maternal care package: a guide to field healthcare workers. Family Health Bureau 2011. https://medicine.kln.ac.lk/depts/publichealth/Fixed_Learning/clearkship/3.PHM/maternal_care_package_a_guide_to_field_healthcare_workers_english.pdf. Accessed 17 Mar 2021.

[11] The Central Bank of Sri Lanka. Economics and social statistics of Sri Lanka. 2019. https://www.cbsl.gov.lk/sites/default/files/cbslweb_documents/statistics/otherpub/ess_2019_e.pdf. Accessed 17 Mar 2021.

[12] Department of Census and Statistics. Census of Population and Housing of Sri Lanka, 2011. http://www.statistics.gov.lk/pophousat/cph2011/pages/activities/Reports/District/Galle.pdf. Accessed 17 Mar 2021.

[13] Agampodi SB, Agampodi TC (2013). Antenatal depression in Anuradhapura, Sri Lanka and the factor structure of the Sinhalese version of Edinburgh post partum depression scale among pregnant women. PLoS One.

[14] Huizink AC, Delforterie MJ, Scheinin NM, Tolvanen M, Karlsson L, Karlsson H (2016). Adaption of pregnancy anxiety questionnaire-revised for all pregnant women regardless of parity: PRAQ-R2. Arch Womens Ment Health.

[15] Zimet G, Dahlem N, Zimet S, Farley G (1988). The multidimensional scale of perceived social support. J Pers Assess.

[16] Barker DJP, Hall AJ (1991). Practical epidemiology.

[17] Dadi AF, Miller ER, Bisetegn TA, Mwanri L (2020). Global burden of antenatal depression and its association with adverse birth outcomes: an umbrella review. BMC Public Health.

[18] Fan Q, Long Q, De Silva V, Gunarathna N, Jayathilaka U, Dabrera T (2020). Prevalence and risk factors for postpartum depression in Sri Lanka: a population-based study. Asian J Psychiatr.

[19] Perera ELS (2017). Fertility transition in Sri Lanka: is it a temporary phenomenon?. J Biosoc Sci.

[20] Ball HA, Siribaddana SH, Kovas Y, Glozier N, McGuffin P, Sumathipala A (2010). Epidemiology and symptomatology of depression in Sri Lanka: a cross-sectional population-based survey in Colombo District. J Affect Disord.

[21] Balestrieri M, Isola M, Bisoffi G, Calò S, Conforti A, Driul L (2012). Determinants of ante-partum depression: a multicenter study. Soc Psychiatry Psychiatr Epidemiol.

[22] Yanikkerem E, Ay S, Mutlu S, Goker A (2013). Antenatal depression: prevalence and risk factors in a hospital based Turkish sample. J Pak Med Assoc.

[23] Pinheiro RL, Areia AL, Mota Pinto A, Donato H (2019). Advanced maternal age: adverse outcomes of pregnancy, a meta-analysis. Acta Medica Port.

[24] Sartorius N (2018). Depression and diabetes. Dialogues Clin Neurosci.

[25] Wilson CA, Newham J, Rankin J, Ismail K, Simonoff E, Reynolds RM (2020). Is there an increased risk of perinatal mental disorder in women with gestational diabetes? A systematic review and meta-analysis. Diabet Med.

[26] Rashid A, Mohd R (2017). Poor social support as a risk factor for antenatal depressive symptoms among women attending public antennal clinics in Penang, Malaysia. Reprod Health.

[27] Stewart RC, Umar E, Tomenson B, Creed F (2014). Validation of the multi-dimensional scale of perceived social support (MSPSS) and the relationship between social support, intimate partner violence and antenatal depression in Malawi. BMC Psychiatry.

[28] Roomruangwong C, Kanchanatawan B, Sirivichayakul S, Maes M (2017). High incidence of body image dissatisfaction in pregnancy and the postnatal period: associations with depression, anxiety, body mass index and weight gain during pregnancy. Sex Reprod Healthc.

